# Backpack on Board: Individual Air Monitoring Predicts Prenatal Exposure to PAHs

**Published:** 2008-11

**Authors:** Cynthia Washam

Scientists studying human exposure to air pollutants have traditionally had to rely on data from stations monitoring ambient pollution levels. These stations are unable to account for neighborhood variation of or indoor exposure to pollutants such as tobacco smoke, and thus do not capture personal exposures. An international group of researchers studying pregnant women in Krakow, Poland, found they could accurately predict individual exposures by using data from personal air monitors, allowing the development of a predictive model of exposure that may be generalizable to pregnant women in similar exposure settings **[*EHP* 116:1509–1518; Choi et al.]**. Moreover, they found most of the women’s exposure was to outdoor pollutants that penetrated indoors.

The researchers assessed the exposure of 341 nonsmoking pregnant women to airborne polycyclic aromatic hydrocarbons (PAHs). PAHs are ubiquitous carcinogenic compounds formed by the incomplete burning of wood, coal, oil, and other organic substances. The eight PAHs selected for analysis are associated with a variety of health effects including cancer, developmental abnormalities, and asthma. PAHs are known to cross the placenta and have been demonstrated to pose significant harm to developing fetuses.

Pregnant women were recruited from prenatal clinics in the center and outskirts of the city. Each was given a backpack equipped with an air monitor to wear for a 48-hour period during the second trimester. Before they went to sleep, the subjects placed the device alongside their beds. A subset of 78 women also used the device for 48-hour periods in their first and third trimesters. To account for seasonal variations in pollution, an approximately equal number of women were enrolled each season. Subjects also completed questionnaires about their health and lifestyle, including exposure to secondhand tobacco smoke.

The results revealed that although most of the women spent less than 3 hours a day outdoors, their personal PAH exposure correlated closely with outdoor levels of the pollutants. The data also showed exposure increased significantly during the winter months with levels declining in the summer, appearing to confirm that coal-burning municipal furnaces and industries were the source of most ambient PAHs in the city.

Using data from the monitors and questionnaires, the researchers reported they could accurately predict personal PAH exposure throughout pregnancy based on the outdoor mean PAH concentration at any given month of the year. They note, however, that indoor data are more accurate for assessing short-term (48 hours or less) individual exposure.

## Figures and Tables

**Figure:**
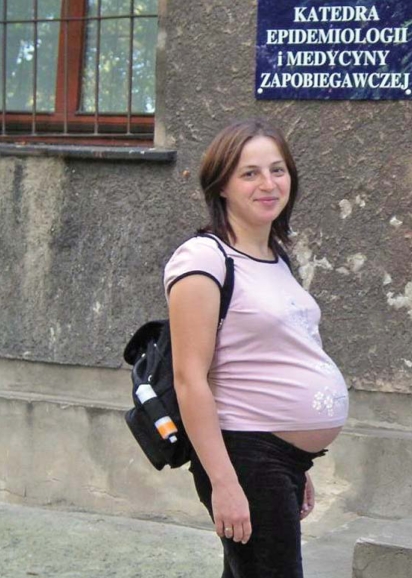
A study of Polish women offers insight into gestational PAH exposure.

